# Kidney Transplant Recipients: Viral Infections and Malignancies

**DOI:** 10.3390/pathogens15040390

**Published:** 2026-04-05

**Authors:** Costin Damian, Adrian Constantin Covic, Ramona Gabriela Ursu, Aida Corina Badescu, Simona Mihaela Hogas, Andreea Simona Covic, Mihai Isache, Silvia Gabriela Ionescu, Corneliu Morosanu, Stefania Brindusa Copacianu, Luminita-Smaranda Iancu

**Affiliations:** 1 Grigore T. Popa University of Medicine and Pharmacy Iasi, 700115 Iași, Romania; costin-damian@umfiasi.ro (C.D.); adrian.covic@umfiasi.ro (A.C.C.); aida.badescu@umfiasi.ro (A.C.B.); simona.hogas@umfiasi.ro (S.M.H.); andreea.covic@gmail.com (A.S.C.); silvia.ionescu@umfiasi.ro (S.G.I.); corneliu.morosanu@umfiasi.ro (C.M.); luminita.iancu@umfiasi.ro (L.-S.I.); 2 Nephrology Clinic, Dialysis and Renal Transplant Center, C. I. Parhon University Hospital, 700503 Iasi, Romania; isache.mihai98@gmail.com; 3 “Cuza-Vodă” Clinical Hospital of Obstetrics and Gynecology, 700038 Iasi, Romania; 4 Clinical Hospital of Infectious Diseases “Sf. Parascheva”, 700116 Iasi, Romania; 5 Microbiology Laboratory, Regional Institute of Oncology, 700483 Iasi, Romania; copacianubrindusa@yahoo.com; 6 National Institute of Public Health, Iasi Regional Center for Public Health, 700465 Iasi, Romania

**Keywords:** kidney transplant, acute kidney injury, BK polyomavirus, cytomegalovirus, Epstein-Barr virus, parvovirus B19, post-transplant malignancy

## Abstract

Kidney transplant recipients (KTRs) remain vulnerable to infectious complications and malignancies due to chronic immunosuppression, both of which may contribute to allograft dysfunction and adverse clinical outcomes. This study aimed to evaluate the prevalence of viral infections and post-transplant malignancies among hospitalized KTRs and to identify factors associated with acute kidney injury (AKI) and chronic graft dysfunction. We conducted a prospective observational study including 215 adult KTRs admitted to a tertiary transplant center over a one-year period. Clinical data, malignancy history, and viral detection for BK polyomavirus (BKV), cytomegalovirus (CMV), Epstein–Barr virus (EBV), and parvovirus B19 were analyzed. AKI occurred in 65.6% of patients, while chronic graft dysfunction was present in 21.4%. Viral positivity was detected in 16.7% of the cohort, most frequently BKV and CMV. Infectious etiologies represented the most common cause of AKI. Viral positivity was significantly associated with infectious mechanisms of AKI and was independently associated with AKI in multivariable analysis (adjusted OR 3.01, *p* = 0.02). In a separate multivariable model, malignancy history (aOR 9.30), viral positivity (aOR 3.33), and concurrent AKI (aOR 3.42) were independently associated with chronic graft dysfunction. These findings suggest that viral reactivation and malignancy history cluster with clinical states of increased graft vulnerability in hospitalized KTRs. Integrated evaluation of infectious, immunologic, and clinical factors may improve risk stratification and management of transplant recipients presenting with acute illness.

## 1. Introduction

Kidney transplantation is the preferred treatment modality for eligible patients with end-stage chronic kidney disease (CKD), conferring significant survival benefits and better quality of life compared to dialysis. Despite progressive improvements in one-year graft survival, which in some international cohorts exceeded 95%, long-term allograft outcomes did not improve at the same rate, with the frequency of graft dysfunction remaining quasi-constant for the past two decades [1]. Many grafts are still compromised by complex, overlapping injury mechanisms [2]. Even if the current clinical paradigm has shifted from the prevention of early acute cellular rejection toward the mitigation of subclinical injuries that erode the functional reserve of the allograft [3], kidney transplant recipients (KTRs) constitute a vulnerable population, frequently requiring hospitalization for both acute and chronic transplant-related pathologies [4].

Hospital presentations in kidney transplant recipients are clinically significant because they frequently represent acute decompensations of a chronically managed, high-risk physiologic state, in which graft function is sensitive to relatively small systemic variations [5]. Numerous presentations happen because of systemic infections, hemodynamic disturbances, medication-related nephrotoxicity, or immune-mediated injury—stressors that sum their effects in real-world inpatient settings and can contribute to kidney functional decline [5,6]. In hospitalized transplant recipients, infectious syndromes are particularly important, because they can induce hemodynamic instability and trigger changes in immunosuppression and antimicrobial drug exposure, which may secondarily influence graft performance [5]. Viral infections remain important to inpatient transplant evaluation because clinically relevant viral replication can be associated with adverse graft-related events, and, for KTRs, infection control and rejection risk must constantly be balanced. Recent international consensus guidance supports the importance of BK polyomavirus (BKV) testing after kidney transplantation and provides updated recommendations for risk stratification and management [7]. Likewise, updated international cytomegalovirus (CMV) consensus guidance continues to highlight this virus as a major transplant pathogen requiring prevention, monitoring, and treatment strategies [8].

Within this inpatient context, two syndromes have important clinical relevance for outcomes: acute kidney injury (AKI) and chronic graft dysfunction. AKI represents an abrupt decline in kidney filtration that can arise from multiple causes, such as perfusion deficits, systemic inflammation, drug effects, obstruction, or immune injury, while chronic graft dysfunction reflects persistent impairment that often signals cumulative structural damage and limited reserves [5,6]. These states are increasingly understood as biologically linked, rather than independent. Recent reviews of the AKI-to-CKD progression explain how acute injury can be followed by dysfunctional repair processes, which promote persistent inflammation and fibrosis, thereby increasing the likelihood of chronic impairment even when the patient shows apparent recovery [9,10]. One dysfunctional repair mechanism involved is the G2/M cell cycle arrest—trigger the secretion of profibrotic and proinflammatory cytokines [11]. This senescence-associated secretory phenotype fosters a microenvironment conducive to fibroblast activation, linking acute ischemic insults directly to long-term fibrosis. In transplant recipients, this linkage is clinically intuitive because acute insults often occur on a background of already present graft vulnerability, and episodes of AKI may accelerate functional decline in an allograft [5,6].

The etiology of AKI in KTRs is distinct from that of the general population, characterized by a complex interplay of calcineurin inhibitor (CNI) nephrotoxicity, ischemia-reperfusion injury (IRI), and infection-driven hemodynamic instability. Recent mechanistic data suggest that AKI episodes serve as a catalyst for accelerated graft dysfunction. Chronic graft dysfunction is described by the Banff classification as interstitial fibrosis and tubular atrophy (IFTA), and represents the final common pathway of allograft loss. Unlike acute rejection, which presents with manifest dysfunction, IFTA progresses insidiously. Its pathogenesis is multifactorial, involving both immunological factors, such as chronic antibody-mediated rejection (cAbMR) and non-immunological insults (hypertension, CNI toxicity, and viral nephropathy). Longitudinal analyses indicate that the rate of estimated glomerular filtration rate (eGFR) decline is often determined by specific, discrete hits to the kidney parenchyma [12]. Identifying the modifiable drivers of this decline—specifically viral coinfections—remains a priority for extending graft longevity.

The obligatory use of maintenance immunosuppression—typically a triple regimen of a CNI (tacrolimus or cyclosporine), an antimetabolite (mycophenolate mofetil), and corticosteroids—creates an immunosuppression environment in which viral replication can occur [13]. While the systemic manifestations of opportunistic infections are well-documented, their specific contribution to renal parenchymal injury is complex. Major viral pathogens that affect kidney transplant recipients reactivate under immunosuppression and contribute to systemic disease or graft dysfunction. Challenges appear in the strategies of viral detection and screening, the differential impacts of each virus on transplant recipients (particularly in the first 6 months post-transplant), and current antiviral prophylaxis and treatment protocols [14].

BK polyomavirus frequently establishes an asymptomatic, latent infection within the human genitourinary tract, specifically within the urothelium, during childhood [15]. Immunosuppression regimens in KTRs can induce reactivation, which occurs in 10–20% of recipients, and in 5–10% of cases leading to BK polyomavirus-associated nephropathy (BKVAN) [16]. The AST-IDCOP guidelines encourage rigorous, prospective monthly screening for BKV DNAemia in the peripheral blood for the first 9 months post-transplant, given the established timeline that viremia reliably precedes destructive structural nephropathy [15].

Cytomegalovirus remains one of the most clinically important and well-studied opportunistic viruses in the field of solid organ transplantation [17], exerting deleterious effects on the allograft [18]. Viral replication is associated with the increase in vascular adhesion molecules (ICAM-1, VCAM-1) on the vascular endothelium, potentially fostering a pro-atherogenic and pro-inflammatory microenvironment [19]. Furthermore, recent studies showed that CMV viremia is an independent risk factor for the development of bacterial and fungal superinfections, especially in immunosuppressed individuals [20,21], which in turn may contribute to sepsis-associated AKI.

Another virus studied in relation to kidney graft injury is the Epstein Barr virus (EBV), with the most severe, life-threatening consequence of uncontrolled EBV infection in KTRs is the development of post-transplant lymphoproliferative disorder (PTLD) [22]. Similarly to the management dilemmas posed by CMV and BKV, the presence of EBV severely complicates clinical decision-making; interventions intended to lower the viral load via immunosuppression reduction must be meticulously and cautiously balanced against the ever-present, countervailing threat of precipitating fatal alloimmunity [23].

Human parvovirus B19 (PVB19) is uniquely characterized by its profound, highly specific tropism for human erythrocyte progenitor cells located within the bone marrow, coupled with a secondary affinity for endothelial cells. In KTRs, the inability to mount a rapid, neutralizing IgG antibody response leads to the uncontrolled viral destruction of erythrocyte progenitors. This manifests clinically as severe, chronic, refractory anemia and pure red cell aplasia, defined hematologically by a profound lack of an appropriate reticulocyte response despite severe anemia [24]. The deleterious effects of PVB19 on the kidney allograft extend also to collapsing glomerulopathy and thrombotic microangiopathy within the transplanted kidney [14].

CKD is increasingly recognized as a pro-carcinogenic state, carrying a significantly increased risk of malignancy, up to an incidence of 10–15%, independent of immunosuppressive medications or transplantation [25]. Large-scale epidemiological data indicate that patients with CKD, particularly those with advanced disease or on dialysis, exhibit higher standardized incidence ratios for cancer compared to the general population, with a specific predilection for urinary tract malignancies (kidney, bladder) and viral-associated cancers [26]. This association appears to be graded; as the (eGFR) declines, the risk of cancer mortality rises, suggesting a direct biological link between uremic toxicity and neoplastic transformation [27]. The demographic of CKD is aging, and consequently, a growing proportion of potential transplant candidates present to selection committees with complex, extensive oncological histories [28]. KTRs exhibit a risk of malignancy that is approximately two- to four-fold higher than that of the age-matched general population, driven by cutaneous and virus-associated cancers [29]. The mechanisms driving this increased susceptibility are likely multifactorial and related to chronic inflammation, oxidative stress, and impaired DNA repair. Furthermore, the accumulation of carcinogenic metabolites and the retention of pro-inflammatory cytokines in renal failure may compromise immune surveillance, mimicking a state of acquired immunodeficiency even before dialysis is initiated [30].

In a recent retrospective cohort study, Ahmed S. et al., linked US transplant and cancer registry data on KTRs treated with belatacept or tacrolimus as the initial maintenance therapy. The overall cancer incidence was found to be 10.1 and 12.6 per 1000 person-years in belatacept and tacrolimus users, respectively, showing that belatacept did not increase cancer risk among KTRs. The study also re-stated that PTLD risk was increased among Epstein-Barr virus-seropositive recipients [31]. Nimmo A et al. recognize that cancer is a major contributor to morbidity following kidney transplantation. Their study examined the incidence of cancer in KTRs from Scotland, compared the general population, and identified factors associated with cancer development. This study utilized data from the Scottish Renal Registry, Scottish Cancer Registry and hospitalization records, between 1997–2021. During this period, 4033 patients aged older than 18 years received a first kidney transplant, and 770 of them developed cancers, much exceeding the 194 expected. Cancer incidence was 7 times greater than that of the general population in KTRs under 40, while for KTRs over 60, this increased incidence was 3 times greater. The authors concluded that they found an increased risk of cancer in KTRs, particularly for younger individuals [32].

The observational COMETA study aimed to elucidate the interplay between the immune system and cancer by integrating comprehensive clinical data with high-throughput small-RNA sequencing on the serum of patients with post-kidney transplant malignancies. The authors analyzed 138 KTRs, and they identified three distinct serum miRNA profiles, respectively, associated with kidney transplantation, pro-oncogenic and onco-protective factors in this unique population. These findings could serve as a basis for future research, paving the way for the development of advanced tools for early cancer diagnosis, precise prognosis formulation, and the creation of targeted therapies for KTRs with neoplastic complications [33].

Aim: KTRs have an increased risk for malignancy and for infections (especially viral reactivation and opportunistic disease) due to chronic immunosuppression. Viruses with kidney tropism can directly affect the allograft, while opportunistic infections are a major cause of hospitalization and mortality. Understanding co-occurrence and associations in real-world hospital presentations can inform screening and management. In this study we aim to estimate the prevalence of prior or current malignancy (post-transplant), and viral infections/reactivations among KTRs presenting to the hospital during a 1-year period. Secondly, we aim to identify factors associated with viral infection, opportunistic infections, allograft dysfunction at presentation or during admission.

## 2. Materials and Methods

Our study included 215 renal transplant patients registered at the Clinical Hospital “Dr. C. I. Parhon”, Iași, Romania, between February 2025 and January 2026. Adult kidney transplant recipients presenting to the study center within the predefined study interval were screened for eligibility. All KTRs who accepted to be part of the study were included, excluding patients under 18 years of age or unable to consent. Inclusion criteria consisted of: (1) prior kidney transplantation and (2) hospital evaluation within the study period. No restrictions were applied based on time since transplantation, indication for admission, or baseline graft function. Participants consented both to the analysis of routinely collected clinical information and, where testing for renal-tropic viral infections had been performed as part of standard clinical care, for the study team to conduct independent retesting of those samples for research purposes.

Patients were included consecutively during the study period, and each patient was included only once. Repeat hospital admissions were not analyzed, and no patient was counted more than once in the dataset. AKI was defined according to KDIGO criteria, based on an increase in serum creatinine relative to baseline values. Chronic graft dysfunction was defined based on biopsy-confirmed graft pathology, as documented in the medical records, which was corroborated with other clinical data and the patient’s medical history, including a history of eGFR decline. For patients presenting with AKI, viral positivity was considered following the onset of AKI; therefore, viral positivity was assessed after the diagnosis of AKI and reflects findings obtained during the clinical workup rather than preceding events.

Total DNA extraction was performed on 400 μL of blood, using the croBEE 201a Nucleic Acid Extraction Kit (GeneProof, Brno, Czech Republic) and the automated croBEE NA16 Nucleic Acid Extraction System (GeneProof, Brno, Czech Republic). The purified DNA was tested for the simultaneous detection of BKV, CMV and EBV have been tested by triple viral detection by quantitative polymerase chain reaction (qPCR), using the genesig^®^ PLEX kit (PrimerDesign, Chandler’s Ford, UK), and PVB19 was tested using a genesig^®^ Advanced Kit from the same manufacturer. The platform used for the qPCR test was the Stratagene MX3005P qPCR platform (Agilent Technologies, Santa Clara, CA, USA). The full experiment description was presented previously [34].

Statistical analysis was performed using IBM SPSS Statistics version 26.0 (IBM Corp., Armonk, NY, USA). All variables were classified a priori as either categorical or continuous. Continuous variables (age) were assessed for distributional properties and were summarized primarily using medians and interquartile ranges (IQR), given their non-Gaussian distribution in hospitalized transplant cohorts. For expanded descriptives, means with standard deviations and ranges were additionally reported to provide full distributional context. Categorical variables were summarized as counts and percentages, with explicit denominators reported for clarity. Viral variables were handled both individually (BK virus, CMV, EBV, parvovirus B19) and as a composite viral positivity variable, defined as positivity for at least one tested virus. Malignancy was treated as a binary variable (present vs. absent), irrespective of cancer type, to preserve statistical power and align with the study’s risk-stratification focus.

A composite variable (“any viral positivity”) was constructed to indicate the presence of at least one detected viral infection (BKV, CMV, EBV, or PVB19). This variable was used as an exploratory measure to capture overall viral detection in the cohort, acknowledging the biological heterogeneity among the included viruses.

Univariate associations between predefined exposures (donor type, malignancy, viral positivity) and outcomes (acute kidney injury and chronic graft dysfunction) were evaluated using Fisher’s exact test for categorical variables. Fisher’s exact test was selected over χ^2^ testing because several exposure–outcome combinations involved small cell counts, particularly for viral and malignancy variables, where χ^2^ assumptions may not hold. Odds ratios (ORs) were calculated for all binary exposure–outcome pairs. When zero counts were present in any contingency table cell, a Haldane–Anscombe correction (addition of 0.5 to all cells) was applied to allow stable OR estimation without inflating statistical significance. Continuous age comparisons between outcome groups were assessed using the Mann–Whitney U test, reflecting non-normal distributions.

A two-sided *p*-value < 0.05 was considered statistically significant for univariate analyses. 

## 3. Results

The cohort comprised 215 kidney transplant recipients admitted to hospital over one year. 65.6% (141/215) met criteria for acute kidney injury (AKI) at presentation and 21.4% (46/215) had chronic graft dysfunction. Cadaveric donor transplantation accounted for 56.7% (122/215) of grafts, and malignancy was documented in 6.5% (14/215). Composite viral positivity (BK and/or CMV and/or EBV and/or parvovirus B19) was present in 16.7% (36/215) of patients. BKV was positive in 19 (8.8%), CMV in 18 (8.4%), EBV in 4 (1.9%), and PVB19 in 1 (0.5%) (Table 1). Viral positivity was identified in a clinically important minority of patients, with composite viral positivity present in 16.7% of the cohort. Malignancy was documented in 6.5% of patients. Among patients without AKI, 28 out of 74 (37.8%) underwent viral testing.

Of the 141 cases of AKI, 57 (40.4%) had an infectious cause (19.1% urinary tract infection (UTI), 14% sepsis, 5% CMV infection, 3.5% BKV nephropathy and 2.8% both CMV and BKV infection), 46 (32.6%) had an immunologic cause (autoimmune rejection 22.7%, inadequate immunosuppression 9.9%) and 38 (27%) of AKI presentation had a hemodynamic or functional cause (dehydration 22% and other cardiovascular causes 5%) (Table 2).

Composite viral positivity differed significantly across AKI mechanisms (Table 3). Viral detection was most frequent among patients with infectious AKI, whereas viral positivity was uncommon in hemodynamic/functional AKI. Overall differences between mechanisms were statistically significant (*p* = 0.0016). Pairwise comparisons demonstrated that viral positivity was more likely in infectious AKI compared with hemodynamic/functional AKI. A similar but less pronounced pattern was observed for immunologic AKI. No statistically significant difference in viral positivity was identified between infectious and immunologic mechanisms.

Expanded descriptive statistics demonstrated that patients with chronic graft dysfunction had a markedly higher prevalence of malignancy, viral positivity, and concurrent acute kidney injury compared with those without chronic dysfunction. Viral testing was more frequently performed among patients with chronic graft dysfunction, reflecting real-world diagnostic practice (Table 4).

In univariate analyses, composite viral positivity was associated with higher odds of AKI (OR 2.56, *p* = 0.05) and was strongly associated with chronic graft dysfunction (OR 3.97, *p* = 0.002). Malignancy was not associated with AKI but was associated with chronic graft dysfunction (OR 5.72, *p* = 0.003). Donor type was not associated with AKI or chronic graft dysfunction in univariate testing (Table 5).

In multivariable logistic regression for AKI adjusted for age, gender, donor type, malignancy, and composite viral positivity, viral positivity remained independently associated with AKI (aOR 3.01, 95% CI 1.18–7.70; *p* = 0.021), while age, gender, donor type, and malignancy were not independently associated with AKI. In a second multivariable model for chronic graft dysfunction adjusted for age, gender, donor type, malignancy, composite viral positivity, and AKI, malignancy (aOR 9.30, 95% CI 2.63–32.87; *p* < 0.001), viral positivity (aOR 3.33, 95% CI 1.49–7.45; *p* = 0.003), and AKI (aOR 3.42, 95% CI 1.35–8.68; *p* = 0.01) were independently associated with chronic graft dysfunction, while donor type, age, and gender were not (Table 6). We performed a sensitivity analysis excluding malignancy from the multivariable model, the association between viral positivity and chronic graft dysfunction remained stable (aOR 3.84, 95% CI 1.77–8.33, *p* < 0.001), with no material changes in the estimates for other variables.

A history of malignancy since the transplantation was documented in 14 kidney transplant recipients. The malignancy spectrum was heterogeneous and predominantly comprised solid organ tumors. Renal tumors represented the most frequent category, with three patients reporting renal cell carcinoma and one patient diagnosed with kidney oncocytoma. Cutaneous tumors were also observed, including two cases of basal cell carcinoma and one case of squamous cell carcinoma. Additional solid tumors involved multiple organ systems and included gastric adenocarcinoma, cecal cancer, uterine carcinoma, ovarian cancer, pulmonary invasive adenocarcinoma, and bladder urothelial carcinoma. One case of Kaposi sarcoma was identified, consistent with the recognized spectrum of malignancies associated with chronic immunosuppression. No single malignancy subtype predominated within the cohort.

Other opportunistic infections were uncommon in this patient group, but clinically notable. Four cases of *Cryptosporidium* spp. infection and 2 Varicella-Zoster virus reactivations occurred in our study cohort.

## 4. Discussion

In this cohort of 215 hospitalized kidney transplant recipients, acute kidney injury represented the most frequent clinical presentation, affecting nearly two thirds of patients, while chronic graft dysfunction was observed in approximately one fifth of the cohort. Viral detection was identified in a minority of patients but showed clear clinical relevance, with composite viral positivity present in about one sixth of recipients. Among the detected viruses, BK polyomavirus and cytomegalovirus were the most frequently identified, whereas Epstein–Barr virus and parvovirus B19 were uncommon. Infectious etiologies constituted the largest proportion of AKI presentations, followed by immunologic and hemodynamic causes. Importantly, viral positivity differed significantly across AKI mechanisms and was markedly more common in infectious presentations compared with hemodynamic or functional AKI.

Viral testing in this study was recommended as part of routine clinical care and not systematically across all patients, being more frequently performed in patients with AKI than in those without AKI (57.4% vs. 37.8%), supporting the presence of differential testing based on clinical severity. This introduces a potential detection bias, whereby viral positivity may be disproportionately identified in patients with worse outcomes. As such, the observed associations between viral positivity and graft dysfunction should be interpreted cautiously, as they may partially reflect differences in testing patterns rather than true biological effects. In clinical context, viral positivity may serve as a useful marker of patient vulnerability and clinical deterioration, supporting its role as a pragmatic tool in risk stratification rather than a direct mechanistic driver of graft injury.

The composite viral positivity variable used in this study includes biologically distinct viruses with differing pathophysiological mechanisms and clinical implications. As such, this variable should be interpreted as a marker of overall viral detection rather than a homogeneous exposure. The observed associations may therefore reflect a general state of immunologic vulnerability or increased clinical complexity rather than the effect of a specific viral pathogen.

In univariate analyses, viral positivity was associated with higher odds of both AKI and chronic graft dysfunction, while malignancy was specifically associated with chronic graft dysfunction. Multivariable logistic regression confirmed that viral positivity remained independently associated with AKI, and that malignancy, viral positivity, and AKI were independent predictors of chronic graft dysfunction. Together, these findings indicate that hospitalized kidney transplant recipients frequently present with acute graft dysfunction and that viral reactivation and prior malignancy cluster with clinical states characterized by increased graft vulnerability.

Sensitivity analysis excluding malignancy from the model did not significantly alter the estimates for other variables, suggesting that the overall model was stable. However, the magnitude of the association between malignancy and chronic graft dysfunction should be interpreted with caution due to the limited number of events.

The present study describes the clinical profile of KTRs evaluated in a hospital setting, a population in which acute complications and graft-related events are more frequent. AKI represented a major clinical feature within the cohort, which is consistent with prior reports demonstrating that kidney transplant recipients experience frequent episodes of acute graft dysfunction that require hospital admission [35]. In contrast to stable outpatient transplant populations, hospitalized recipients are disproportionately affected by hemodynamic instability, infectious processes, drug-related nephrotoxicity, and immunologic injury, all of which are well-recognized drivers of AKI [36].

Our analysis of kidney transplant recipients presenting to hospital over one year highlighted three clinically relevant and statistically independent contributors to chronic graft dysfunction at presentation: malignancy history, viral positivity, and concurrent AKI. The magnitude and consistency of these associations suggest chronic graft dysfunction in hospitalized KTRs clusters in a subgroup characterized by cumulative immunologic and clinical vulnerability. Malignancy may indicate a longer period since transplantation, more intensive or prolonged immunosuppression, and/or downstream effects of cancer therapies or immunosuppression modifications, all of which may predispose to chronic allograft injury and destabilization during acute illness.

A notable finding in our dataset is that composite viral positivity was not only associated with chronic graft dysfunction but also independently associated with AKI after adjustment for donor type, malignancy, and demographics. This supports the clinical observation that viral reactivation and renal-tropic viral infections may contribute directly to acute graft dysfunction and may also serve as a marker of heightened net immunosuppression. Because viral testing is often clinically triggered, these results should be interpreted as associations within a real-world diagnostic context rather than as population-level prevalence estimates. Donor type (cadaveric versus living) was not associated in our cohort neither with AKI, nor with chronic graft dysfunction in univariate or adjusted analyses. This suggests that, among hospitalized presentations spanning various post-transplant timeframes, clinical factors and immune status may outweigh donor source in determining acute and chronic graft risk. Age and gender similarly showed no independent association with either outcome in these models.

The predominance of infectious mechanisms observed among AKI cases is concordant with existing transplant literature. Chronic immunosuppression results in impaired host defense, predisposing recipients to bacterial, viral, and opportunistic infections that may impair allograft function [37]. Infectious complications remain one of the leading causes of hospitalization after kidney transplantation, with urinary tract infections and systemic infections frequently implicated. Infection-associated AKI may arise through multiple mechanisms, including systemic inflammatory responses, altered renal perfusion, tubular injury, and virus-mediated cytopathic effects [35,38].

Interpretation of these descriptive findings must consider the study context. Hospital-evaluated transplant cohorts differ fundamentally from registry-derived or outpatient populations, as they represent patients experiencing acute clinical deterioration [35]. Consequently, the relative frequencies of AKI, infectious etiologies, and viral detection observed in this study may exceed those reported in broader transplant surveillance datasets. Such differences are methodologically expected and reflect the clinical realities of hospitalized transplant recipients rather than sampling bias [39].

Królicki T et al. mentioned that AKI in transplant patients is a common, yet poorly investigated, complication of urinary tract infections and sepsis starting from the urinary tract. This retrospective study included 101 KTRs with urosepsis, 100 KTRs with UTI, and 100 KTRs with no prior history of UTI or sepsis. AKI incidence in the urosepsis group was 75.2%, while the UTI group had an incidence of 41%. The sepsis patients also had presented with a significantly higher prevalence of AKI stage 2 and 3 compared to the UTI group. In KTRs with both urosepsis and UTI, the occurrence of AKI signals poor transplantation outcomes. The study found the local transfusion policy, modulation of immunosuppression and stress ulcer prophylaxis to be possible modifiable factors that can significantly impact the outcome of the transplant, in the long term [40].

Sato B et al., aimed to identify risk factors for sepsis-associated AKI and estimate the impact of sepsis on graft function in KTRs. Their study was a retrospective cohort evaluation, including 282 KTRs with sepsis admitted to the intensive care unit, finding an AKI rate of 83%, with 35.5% requiring renal function replacement therapy (RRT). Of these requiring RRT, 38 patients survived, with 30 recovering graft function, and 8 remained on RRT. Mortality after 3 months post-ICU admission was 39.7%, with 6.0% experiencing allograft loss. The authors concluded that sepsis-associated AKI is a common complication in KTRs admitted to the ICU, with a high rate of RRT requirement, that is influenced by baseline renal function. The authors also state that despite the severity, in some survivors, graft function may recover even among those with severe AKI [41].

The AKI frequency observed in our study falls within this spectrum, reflecting a hospitalized population with heterogeneous clinical severity. The progressive increase in AKI frequency across studies of urinary tract infection, urosepsis, and ICU-level sepsis suggests that the burden of graft dysfunction is closely linked to the severity of systemic illness.

The association between infectious presentations and viral detection observed in this study highlights the role of viral pathogens in transplant outcomes. BKV and CMV are associated with adverse long-term outcomes [42,43]. Viral replication may contribute to renal injury through direct cellular damage, immune activation, and amplification of inflammatory pathways [42]. Importantly, viral infections frequently coexist with other clinical stressors, complicating causal attribution during acute hospital presentations [37]. BK polyomavirus positivity observed in this cohort is consistent with the well-established tropism of BKV for the renal allograft. Although the number of BK-positive patients was limited, the detection of BKV among hospitalized recipients supports the concept that viral reactivation remains a clinically relevant phenomenon in immunosuppressed transplant populations. Given the multifactorial nature of graft dysfunction, BKV detection should be interpreted within the broader clinical context rather than as an isolated determinant of AKI.

Zhou X et al. noted that BKV is mainly linked to BKV-associated nephropathy in kidney transplant patients and hemorrhagic cystitis, though the mechanisms of BKV latency and reactivation remain unclear and underexplored. This research team highlighted recent progress in understanding these mechanisms, as well as BKV-related diseases and tumorigenesis. The authors emphasize BKV’s key role in potential tumorigenesis, stressing the need for further research into its etiology and pathogenicity both in vitro and in vivo [44].

Wychera C et al. investigated the link between BK viremia and changes in eGFR in children during the first year after allogeneic hematopoietic cell transplant (HCT). Plasma BK viral load was measured by qPCR at weeks 4–7 and 10–13 post-transplant in 136 patients aged under 26 years old. The rate of BKV detection reached 40% of patients at the initial test, and 46% in the second test, with a notable proportion having viral loads of over 10,000 copies/mL. The authors found that the high BK viral loads detected were significantly associated with reduced eGFR at one year post-HCT [45].

Cannon E et al. reported that KTRs are three to four times more likely to develop urothelial carcinoma (UC) than the general population, and studied the link between UC and BKV infection, which affects around 15% of this patient group. Their review identified fourteen KTRs who developed UC after transplant, out of which ten had a history of BKV infection, and six of those developed a rare micropapillary UC subtype, which typically occurs in less than 1% of cases. All micropapillary tumor samples tested positive for the SV40 antigen. The authors suggested that BKV may play a pathogenic role in the development of these rare tumors and high-grade UC, in patients with kidney transplant with prior BK viremia [46].

A review of 41 studies on bladder cancer following kidney transplantation found significant variation in prevalence, diagnosis timing, cancer type, and survival rates. The authors reported on a small study of 103 renal transplant patients, which found no connection to bladder cancer, and a large 1886 patient study showed a strong link, identifying BKV (RR = 11.7) and smoking (RR = 5.6) as significant risk factors for this malignancy. This review concluded that factors such as carcinogen exposure, BKV, HPV, immunosuppressants, and immunosuppression increase bladder cancer risk after transplantation. mTOR inhibitors may lower this risk, and reducing immunosuppression is recommended if cancer occurs [47].

Cohen-Bucay A et al. highlight that BKV reactivation remains a serious concern in immunosuppressed patients. Traditionally, the virus has been recognized primarily as the cause of BKV-associated nephropathy and allograft failure in kidney transplants. However, researchers note that recent studies indicate it also negatively affects native kidney function, reduces patient survival in other types of organ transplants, and may even contribute to other conditions such as cancer. Because viral pathogenesis is driven by a weakened immune system, the standard clinical approach involves reducing the patient’s immunosuppressive therapy. The authors caution that this strategy carries significant risks, as lowering immunosuppression can lead to the development of donor-specific antibodies that threaten the long-term viability of the transplanted organ. Ultimately, they emphasize that there is a significant gap in clinical care due to the absence of targeted antiviral medications, arguing that the complex management and high morbidity associated with the disease necessitate the development of dedicated anti-BKV therapies [48].

In a comprehensive review, Manole B et al., performed a systematic search of the PubMed and EMBASE databases, which was carried out for all the published studies on renal cell carcinoma (RCC) in from the 1 January 2021 to the 1 May 2022, using the following search algorithm: RCC and urothelial carcinoma, and oncogenic viruses (BKV, EBV, HCV, HPV and Kaposi Sarcoma Virus), RCC and biomarkers, immunohistochemistry (IHC). The virus found to be most frequently associated with RCC was BKV. The authors considered this relation to be important, as a virus-induced tumor could efficiently benefit from preventive vaccination or by targeted therapies [49].

The prevalence and diversity of malignancies within the cohort align with the recognized oncologic burden of transplant recipients. Long-term immunosuppression is strongly associated with elevated cancer risk, particularly for skin cancers, virus-related malignancies, and certain solid tumors [50,51]. The heterogeneous distribution of malignancy types observed here mirrors findings from registry-based analyses, where no single tumor category was predominant [50]. Kaposi sarcoma, although uncommon, remains a characteristic malignancy associated with immunosuppressed states and has been repeatedly described in transplant populations [52].

The use of valganciclovir (VGC) for prophylaxis of CMV infection is often limited by drug-related adverse effects and the need for dose adjustments due to declining renal function. Trappe M et al. conducted a retrospective analysis of CMV viremia episodes within the first year post-transplant in a cohort of 316 recipients to identify factors associated with persistent infection. Their findings indicated that nearly all patients exhibited a high-risk (HR) profile for CMV infection. CMV viremia was detected in 22% of the overall cohort, with the HR subgroup being most affected (showing a prevalence of 44.1%). The analysis demonstrated that the occurrence of viremia during ongoing antiviral prophylaxis is associated with an increased risk of sustained viral replication and development of antiviral resistance in HR individuals. Furthermore, a lower eGFR and reduced VGC dosing after transplant were identified as contributing risk factors for breakthrough infections among HR patients in this single-center study. The authors also stated that these patients may benefit from enhanced CMV surveillance or alternative prophylactic strategies such as letermovir [53].

Thrombotic microangiopathies are uncommon, but potentially serious in KTRs. Von Tokarski F. et al. studied this condition in 1644 KTRs from 2009 to 2021, finding a 4.7% incidence, accounting to 77 cases. Identified triggers included EBV/CMV (10 cases) and bacterial infections (6 cases). The study noted that thrombosis management varies and timing influences incidence, causes, and outcomes [54].

Kumar L. et al. conducted a systematic review and meta-analysis of preemptive therapy (PET) with weekly CMV polymerase chain reaction monitoring for ≥3 months and universal prophylaxis (UP) with 6 months of valganciclovir. The authors considered that PET is associated with a significantly lower incidence of CMV disease compared to UP with similar rates of other clinical outcomes. Their findings provide rationale and preliminary data for a randomized superiority trial of optimized PET versus UP in donor seropositive recipient seronegative kidney transplant recipients [55]. Ishiyama K et al. recognized that CMV infection is associated with graft rejection in renal transplantation. The authors studied the in vivo kinetics of lymphocytes in CMV-infected renal transplant patients using longitudinal samples compared with those of nonviremic patients. They identified that all viremic patients, with one exception, overcame viremia and did not experience graft rejection. This paper provides insights into the in vivo dynamics and interplay of cytotoxic lymphocytes responding to CMV viremia, which are potentially linked with control of CMV viremia to prevent graft rejection [56].

Di Cristanziano V et al. reported that treatment options for CMV infections in immunosuppressed patients are limited, mainly consisting of VGC/ganciclovir (GCV) as the first-line treatment. In this small case series, combined therapy with CMV-specific IVIg, letermovir, and VGc/GCV was associated with sustained virological control in transplant recipients with complicated CMV infection unresponsive to standard therapy, suggesting a potential role for multi-modal antiviral and immunomodulatory strategies in this high-risk setting. In patients with prolonged CMV viremia, treatment with a combined therapy based on letermovir, CMV-specific intravenous immunoglobulins, and VGC/GCV, which led to the sustained control of CMV viremia in all presented cases [57].

A study by Donia AF et al., analyzed de novo malignancy as a complication after kidney transplantation. The type of cancer may vary due to factors such as the prevalence of viral infection and race, with Kaposi sarcoma being the most common malignancy, in the past, among transplanted patients, constituting more than one-third of cancers. The authors analyzed data of 3126 KTRs, which was retrieved and retrospectively analyzed. Since 2010, no new cases of Kaposi sarcoma have been observed in the analyzed cohort. Patients who received CMV prophylaxis and/or were maintained on mTOR inhibitor or steroid-free regimens have not developed Kaposi sarcoma. The authors concluded that Kaposi sarcoma, which was previously the most common malignancy, is no longer observed for almost a decade among our kidney transplant recipients, m-TOR inhibitors, steroid-free regimen and CMV prophylaxis policy are possible contributing factors [58]. In our patient group, only one case of post-transplant Kaposi sarcoma appeared, which may also be interpreted as a rare occurrence of this malignancy type.

Désy O et al. studied the role of infections and cancer as major causes of premature death in organ recipients. The authors previously reported that a cell-based assay measuring CD14 + 16 + tumor necrosis factor-α + monocytes after peripheral blood mononuclear cell (PBMC) incubation with Epstein-Barr virus peptides has a high sensitivity for detecting over-immunosuppression (OIS) events in kidney recipients, in the short term. In the currently reported research, the authors aimed to develop a risk score for predicting long-term events and they studied 551 PBMC samples from 118 kidney recipients. The patients were followed for a median of 6 years. Of these, 40 (34%) experienced an OIS event. The authors concluded that using a combination of age and in vitro PBMC response to Epstein-Barr virus peptides allows a substantial shift in the estimated risk of OIS events [59].

Díez-Vidal A et al. investigated EBV’s role in the evolution of complications KTRs. The team focused on two cases of EBV-induced acute colitis in transplanted patients. KTRs with gastric-limited disease were typically healthy women and exhibited a benign course, characterized by a more acute presentation and complete recovery in all cases. In contrast, patients with intestinal disease were often immunocompromised, presenting with deep colonic ulcers frequently associated with rectal bleeding, high rates of perforation, frequent need for surgical intervention, and significant mortality. The paper concluded that EBV-related gastrointestinal disease varies by patient immunocompetence and the site of involvement, underlining that individualized treatment strategies and vigilant long-term follow-up are needed due to the lack of standardized treatment protocols and the risk of relapse or development of EBV-associated lymphoproliferative disorders [60].

Jiang X et al. state that KTRs benefit from the best treatment for patients with end-stage renal disease, but still face important postoperative complications during the recovery period. PTLDs represent one of the severe and life-threatening complications that occur after transplant, while the recipient is undergoing immunosuppressive therapy, and the recognized risk factors for this conditions include EBV infection, the cumulative degree of immunosuppression, as well as genetic aspects. The authors present a 41-year-old male KTR which was diagnosed with EBV-associated T/NK cell monomorphic PTLD, by a blood EBV DNA test, pharyngeal biopsy, and corresponding pathological examination. Their laboratory tests and clinical symptoms normalized after six cycles of chemotherapy. For PTLD, discontinuing immunosuppression and starting systemic treatment can help in disease regression, but, since the incidence of this disease is low, management is limited by the scarcity of clinical experience and limited data [61].

In a multicenter study conducted in the USA, Tajima T. et al. conducted another study on EBV-associated PTLD in pediatric patients after transplant. The study enrolled 944 patients younger than 21 years of age. Of these, 872 received liver, heart, kidney, intestinal, or multivisceral transplants in seven US centers between 2014 and 2019. In total, 34 pediatric EBV+ PTLD (3.9%) were identified by biopsy. The study was in line with retrospective findings, that donor positive, recipient negative (D + R−) serostatus constitutes a significant risk factor for the development of PTLD, being strongly correlated monomorphic and polymorphic PTLD subtypes. Patients who developed monomorphic or polymorphic PTLD exhibited a higher incidence and burden of EBV DNAemia compared with transplant recipients who did not develop PTLD. The authors concluded that rigorous monitoring of EBV viral load during the early post-transplant period is warranted, particularly in pediatric recipients with D + R− serostatus and in non-liver transplant recipients aged five years or older at the time of transplantation [62].

Chiodo Ortiz A. et al. analyzed 354 adults who underwent kidney only transplantation from January 2015 through September 2021 at one medical center. Patients underwent treatment with either low-doses of mycophenolate, tacrolimus and sirolimus or low-doses of mycophenolate, tacrolimus and belatacept. All recipients underwent induction with antithymocyte globulin and a rapid glucocorticosteroid taper. Relevant donor and recipient information were analyzed and endpoints of PTLD were assessed. There were no cases of PTLD in either cohort within the study period, supporting that non-belatacept (MMF, tacrolimus and sirolimus) and belatacept-based (MMF, tacrolimus and belatacept) regimens do not appear to pose any increased risk of early onset PTLD. Both cohorts benefited from low rates of rejection, malignancy, mortality and graft failure [63].

PVB19 positivity was rare in our cohort, consistent with its lower overall prevalence compared to CMV and BKV in adult transplant populations. Nevertheless, given its potential to induce refractory anemia and, in some cases, graft dysfunction, its detection remains clinically meaningful. The limited number of positive cases in this study precludes formal analysis of outcome associations, but the inclusion of PVB19 within multiplex viral screening reflects an integrated approach to evaluating unexplained cytopenias or graft instability in immunosuppressed recipients.

Hegde UN et al., analysed by PCR, PVB19 viremia, in 21 patients undergoing kidney transplantation between 2013 and 2022. Prevalence of PVB19 disease was 1.9% (21/1164) with a median onset time of 39 days post transplantation. The most frequent clinical symptoms were fatigue (76% of patients), followed by fever (47%), myalgia (33%), and dyspnea (23%). All patients developed anemia, while leukopenia and thrombocytopenia were observed in 14% and 9.5% of patients, respectively, and 61.9% of the infected patients suffered from graft dysfunction. The authors concluded that PVB19, while uncommon, can be a significant cause of refractory anemia, particularly within the first-year posttransplant. Diagnosing PVB19 infection with PCR is crucial, and the primary treatment involves reducing immunosuppressants, especially antiproliferative agents [64].

A case report of a 32-year-old male KTR with PVB19-associated anemia, was published by Aghaei M et al. The patient was admitted to the hospital with anemia, 2 months after transplantation, with symptoms of fever, weakness, palpitations, and marked fatigue. Laboratory evaluation demonstrated severe normocytic anemia, with a hemoglobin level of 5 g/dL and stable creatinine (1.41 mg/dL). Despite transfusion of leukocyte-reduced packed red blood cells, the anemia persisted, raising suspicion for alternative etiologies. Virological screening was subsequently performed, and serum PCR testing confirmed PVB19 DNA positivity, leading to the discontinuation of mycophenolate mofetil and intravenous immunoglobulin administration. Although hemoglobin levels modestly improved to 7 g/dL, the patient was readmitted with pancytopenia and deteriorating graft function. Ultimately, graft nephrectomy was performed due to irreversible rejection [65].

Zhang QQ et al. assessed the clinical routine monitoring methods of PVB19 recipients to allow modulation of immunosuppression. In their retrospective study, the authors evaluated the utility of the function and numbers of lymphocyte subsets in monitoring PVB19 infections in renal recipients posttransplant. The PVB19 infected group, consisting of 37 patients, had significantly lower absolute counts and functions of different lymphocyte subsets compared with immune-stable recipients. The authors recommended prospective risk stratification for the high-risk population at risk of early-onset PVB19 infection, and its recurrence involves screening strategies of immune-based surveillance [66].

Inoue D et al. reported a case of PVB19-associated pure red cell aplasia (PVB19-PRCA) appearing in a patient who received a kidney allograft. The graft originated from a living-donor, at the age of 60, and despite successfully reaching a normal creatinine level, the recipient developed severe anemia that required frequent blood transfusions 2 months after transplantation. The diagnosis was established to be PVB19-PRCA, and transmission of PVB19 through the allograft was confirmed. Their case demonstrates that donor-transmitted PVB19 infection should be suspected in kidney transplant recipients who develop refractory anemia during the early post-operative phase [67].

The independent associations observed between viral positivity, AKI, and chronic graft dysfunction support a model in which acute infectious or inflammatory insults act upon a vulnerable allograft substrate characterized by reduced physiologic reserve. In this context, AKI may represent both an immediate clinical manifestation and a mechanistic bridge toward longer-term structural decline. Viral replication, whether causative or reflective of net immunosuppression, appears to cluster within this high-risk phenotype. These findings reinforce the concept that graft dysfunction in hospitalized KTRs is rarely attributable to a single isolated factor, but rather is a result of immunologic, infectious, and hemodynamic stressors.

Limitations: This study’s strengths include the capture of a relatively large cohort of hospital-presenting kidney transplant recipients over a defined interval and evaluation of intersecting exposures (viral infection, malignancy, donor type) against clinically meaningful outcomes. Limitations include the observational design, low counts for rarer viruses, and the likelihood of clinically driven testing and ascertainment, which may bias associations toward patients with graft dysfunction or systemic illness. Prospective studies with standardized testing, incorporation of transplant vintage, and medication-level immunosuppression measures would help refine causal pathways and actionable risk stratification.

Regarding statistical analysis, several determinants of graft outcomes, including time since transplantation, immunosuppressive regimen and intensity, and prior rejection history, were not available for inclusion in the analysis. These variables are closely linked to both viral reactivation and malignancy risk, and their omission may result in residual confounding. In particular, higher levels of immunosuppression may predispose to both viral positivity and adverse graft outcomes, potentially inflating the observed associations. Therefore, the findings of this study should be interpreted with caution, as the regression models may be under-adjusted.

## 5. Conclusions

In this prospective observational cohort of kidney transplant recipients evaluated at hospital presentation, acute kidney injury emerged as a dominant clinical feature, reflecting the vulnerability of the renal allograft during episodes of acute illness. Infectious mechanisms represented the most frequent contributors to AKI, consistent with the recognized susceptibility of immunosuppressed transplant recipients to infectious complications. Composite viral positivity demonstrated a strong association with infectious presentations of AKI, supporting the concept that viral detection frequently accompanies clinically significant events in hospitalized transplant populations. Viral positivity was also associated with chronic graft dysfunction, suggesting that viral replication may represent an important marker of graft vulnerability.

A prior history of malignancy was observed in a subset of recipients and reflected a heterogeneous spectrum of neoplastic disease, consistent with the expected oncologic burden of long-term transplant survivors. While malignancy was not a primary determinant of acute presentations, its presence highlights the complex clinical background characteristic of transplant populations.

These findings highlight the multifactorial nature of graft dysfunction in real-world clinical settings and underscore the importance of integrated evaluation of infectious, hemodynamic, and immunologic factors in transplant recipients presenting to hospitals. Further studies with larger cohorts are warranted to clarify the mechanistic relationships between viral infections and graft outcomes.

## Figures and Tables

**Table 1:** Baseline characteristics of the study population.

Characteristic	Value
Patients, *n*	215
Age, median (IQR)	48 (40–56)
Male gender, *n* (%)	123 (57.2%)
Female gender, *n* (%)	92 (42.8%)
Cadaveric donor, *n* (%)	122 (56.7%)
Living donor, *n* (%)	93 (43.3%)
AKI (primary outcome), *n* (%)	141 (65.6%)
Chronic graft dysfunction (secondary outcome), *n* (%)	46 (21.4%)
Malignancy present, *n* (%)	14 (6.5%)
BK positivity, *n* (%)	19.0 (8.8%)
CMV positivity, *n* (%)	18.0 (8.4%)
EBV positivity, *n* (%)	4.0 (1.9%)
Parvovirus B19 positivity, *n* (%)	1.0 (0.5%)
Any viral positivity (composite), *n* (%)	36 (16.7%)

**Table 2:** Causes of AKI presentations.

AKI Cause	*n*	% of AKI Cases
Autoimmune	32	22.70%
Dehydration	31	22.00%
UTI	27	19.10%
Sepsis	14	9.90%
Inadequate immunosuppression	14	9.90%
CMV Infection	7	5.00%
Cardiovascular	7	5.00%
BKV Nephropathy	5	3.50%
BKV + CMV Infection	4	2.80%

**Table 3:** Viral positivity across AKI mechanisms.

AKI Mechanism	Viral Negative *n* (%)	Viral Positive *n* (%)
Hemodynamic/Functional	37 (97.4%)	1 (2.6%)
Infectious	38 (66.7%)	19 (33.3%)
Immunologic	36 (78.3%)	10 (21.7%)
Pairwise Comparisons		
Comparison	Odds Ratio	*p*-value
Infectious vs. Hemodynamic	18.5	<0.001
Immunologic vs. Hemodynamic	10.3	0.01

**Table 4:** Expanded descriptive parameters stratified by chronic graft dysfunction.

Characteristic	Total	Chronic Yes	Chronic No
Age (years): mean ± SD; median (IQR); min–max	47.3 ± 12.4; 48.0 (39.5–56.0); 18.0–78.0	45.8 ± 11.8; 47.0 (40.2–53.0); 19.0–72.0	47.7 ± 12.6; 48.0 (39.0–56.0); 18.0–78.0
Male gender	123/215 (57.2%)	25/46 (54.3%)	98/169 (58.0%)
Cadaveric donor	122/215 (56.7%)	27/46 (58.7%)	95/169 (56.2%)
Malignancy present	14/215 (6.5%)	8/46 (17.4%)	6/169 (3.6%)
Any viral positivity (composite)	36/215 (16.7%)	16/46 (34.8%)	20/169 (11.8%)
AKI (primary outcome)	141/215 (65.6%)	38/46 (82.6%)	103/169 (60.9%)
BK tested	108/215 (50.2%)	34/46 (73.9%)	74/169 (43.8%)
BK positive (of tested)	19/108 (17.6%)	10/34 (29.4%)	9/74 (12.2%)
CMV tested	109/215 (50.7%)	34/46 (73.9%)	75/169 (44.4%)
CMV positive (of tested)	18/109 (16.5%)	9/34 (26.5%)	9/75 (12.0%)
EBV tested	13/215 (6.0%)	5/46 (10.9%)	8/169 (4.7%)
EBV positive (of tested)	4/13 (30.8%)	1/5 (20.0%)	3/8 (37.5%)
PVB19 tested	8/215 (3.7%)	3/46 (6.5%)	5/169 (3.0%)
PVB19 positive (of tested)	1/8 (12.5%)	1/3 (33.3%)	0/5 (0.0%)

**Table 5:** Univariate associations (Fisher exact for binary; Mann–Whitney for age).

Exposure	Outcome	Event Rate (Exposed)	Event Rate (Unexposed)	OR	*p*
Cadaveric donor	AKI	81/122 (66.4%)	60/93 (64.5%)	1.09	0.774
Cadaveric donor	Chronic graft dysfunction	27/122 (22.1%)	19/93 (20.4%)	1.11	0.867
Malignancy	AKI	7/14 (50.0%)	134/201 (66.7%)	0.50	0.247
Malignancy	Chronic graft dysfunction	8/14 (57.1%)	38/201 (18.9%)	5.72	0.003
Any viral positivity	AKI	30/36 (83.3%)	111/179 (62.0%)	3.06	0.013
Any viral positivity	Chronic graft dysfunction	16/36 (44.4%)	30/179 (16.8%)	3.97	<0.001
BK positivity	AKI	15/19 (78.9%)	65/89 (73.0%)	1.38	0.775
BK positivity	Chronic graft dysfunction	10/19 (52.6%)	24/89 (27.0%)	3.01	0.054
CMV positivity	AKI	17/18 (94.4%)	64/91 (70.3%)	7.17	0.038
CMV positivity	Chronic graft dysfunction	9/18 (50.0%)	25/91 (27.5%)	2.64	0.092
Age (continuous)	AKI	median 48.0	median 48.5		0.272
Age (continuous)	Chronic graft dysfunction	median 47.0	median 48.0		0.312

**Table 6:** Multivariable logistic regression models.

AKI (Primary)—Related to Age + Gender + Donor Type + Malignancy + Any Viral Positivity			
Predictor	aOR	95% CI	*p*
Age	0.99	0.96–1.01	0.323
Gender_male	0.93	0.51–1.67	0.800
Donor_cadaver	1.13	0.62–2.06	0.689
Malignancy_bin	0.49	0.16–1.52	0.218
Any_viral_pos	3.01	1.18–7.70	0.022
**Chronic Graft Dysfunction (Secondary)—Related to Age + Gender + Donor Type + Malignancy + Any Viral Positivity + AKI**			
**Predictor**	**aOR**	**95% CI**	***p***
Age	0.98	0.96–1.01	0.309
Gender_male	0.98	0.47–2.04	0.965
Donor_cadaver	1.26	0.59–2.67	0.552
Malignancy_bin	9.30	2.63–32.87	<0.001
Any_viral_pos	3.33	1.49–7.45	0.003
AKI	3.42	1.35–8.68	0.01

## Data Availability

The original contributions presented in the study are included in the article, further inquiries can be directed to the corresponding author.

## Abbreviations

The following abbreviations are used in this manuscript:

AKI	Acute Kidney Injury
AST-IDCOP	American Society of Transplantation Infectious Diseases Community of Practice
BKV	BK Polyomavirus/BK Virus
BKVAN	BK Polyomavirus–Associated Nephropathy
CA	California
cAbMR	Chronic Antibody-Mediated Rejection
CD	Cluster of Differentiation
CKD	Chronic Kidney Disease
CMV	Cytomegalovirus
CNI	Calcineurin Inhibitor
CTLA-4	Cytotoxic T-Lymphocyte Antigen-4
DNA	Deoxyribonucleic Acid
D+/R−	Donor Positive/Recipient Negative
EBV	Epstein–Barr Virus
eGFR	Estimated Glomerular Filtration Rate
Fc	Fragment Crystallizable Region
G1/G2	APOL1 Gene Risk Variants
GCV	Ganciclovir
HCT	Hematopoietic Cell Transplant
HR	High Risk
ICAM-1	Intercellular Adhesion Molecule-1
ICU	Intensive Care Unit
IFTA	Interstitial Fibrosis and Tubular Atrophy
IgG	Immunoglobulin G
IgM	Immunoglobulin M
IHC	Immunohistochemistry
IL	Interleukin
IRI	Ischemia-Reperfusion Injury
IVIg	Intravenous Immunoglobulin
IQR	Interquartile Range
KTR	Kidney Transplant Recipient
miRNA	MicroRNA
MMF	Mycophenolate Mofetil
mTOR	Mechanistic Target of Rapamycin
NK	Natural Killer Cells
OIS	Over-Immunosuppression
OR	Odds Ratio
PBMC	Peripheral Blood Mononuclear Cell
PCR	Polymerase Chain Reaction
PD-1	Programmed Cell Death Protein 1
PD-L1	Programmed Death-Ligand 1
PET	Preemptive Therapy
PTLD	Post-Transplant Lymphoproliferative Disorder
qPCR	Quantitative Polymerase Chain Reaction
RCC	Renal Cell Carcinoma
RNA	Ribonucleic Acid
RRT	Renal Replacement Therapy
SD	Standard Deviation
SV40	Simian Virus 40
UC	Urothelial Carcinoma
UP	Universal Prophylaxis
USA	United States of America
UTI	Urinary Tract Infection
VCAM-1	Vascular Cell Adhesion Molecule-1
VGC	Valganciclovir
